# Long-Term Prognosis, Risk Assessment, and Management of Patients Diagnosed with Takotsubo Syndrome: A Narrative Review

**DOI:** 10.3390/jpm15090425

**Published:** 2025-09-04

**Authors:** Małgorzata Kosek-Nikołajczuk, Ewa Borowiak, Radoslaw Piatkowski, Marcin Grabowski, Monika Budnik

**Affiliations:** 1st Department of Cardiology, Medical University of Warsaw, Stefana Banacha 1a, 02-097 Warsaw, Poland; m.kosek.nikolajczuk@gmail.com (M.K.-N.); ewa.anna.borowiak@gmail.com (E.B.);

**Keywords:** takotsubo syndrome, long-term, outcome, prognosis, recurrence, survival, biomarkers

## Abstract

Takotsubo syndrome (TTS) is a condition marked by sudden and temporary dysfunction of the left ventricle, occurring without significant coronary artery disease. It was previously thought to be a benign and self-limiting condition, associated with a favorable long-term prognosis and minimal impact on survival. However, the most recent findings provide evidence that TTS is a heterogeneous condition with various presentation patterns. Using the most recent evidence regarding long-term prognosis in TTS, this review article aims to provide an overview of the long-term survival of patients with TTS, highlighting potential risk factors and comorbidities that may worsen prognosis. It also explores the risk of recurrence and the utility of advanced imaging modalities for prognosis assessment. Risk factors negatively impacting long-term outcomes include male sex, older age, reduced left ventricular ejection fraction (LVEF), physical triggers (especially pulmonary and neurological diseases), and comorbidities such as atrial fibrillation, chronic obstructive pulmonary disease, and active cancer. Recurrence, though relatively uncommon, can affect up to 11% of patients, with “super recurrence” linked to higher peak troponin levels, lower LVEF, and emotional triggers. Advanced imaging modalities—such as coronary angiography and ventriculography, which are considered the gold standard, along with serial echocardiographic assessment—combined with cardiac biomarkers, including relatively low peak troponin levels and markedly elevated N-terminal pro-B-type natriuretic peptide (NT-proBNP), as well as diagnostic ratios like copeptin/NT-proBNP, provide a robust framework for differentiating TTS from acute coronary syndromes. Key findings suggest that chronic therapeutic strategies in the long-term management of TTS patients should focus on improving long-term outcomes and reducing the risk of mortality and TTS recurrence. Methods: A comprehensive review was conducted using PubMed (U.S. National Library of Medicine and National Institutes of Health) and Google Scholar to identify relevant English-language publications addressing the long-term prognosis, biomarkers, imaging, risk of recurrence, and long-term management of TTS.

## 1. Introduction

Takotsubo syndrome (TTS) is defined as a temporary left ventricular (LV) dysfunction, with LV regional wall hypokinesis extending beyond a single coronary artery distribution and without any significant coronary lesions [1]. This disease was first described by Sato et al. in 1990 in Japan [2]. The name “takotsubo” is derived from the Japanese term for an octopus pot, reflecting the characteristic ballooning of the LV apex observed during acute episodes [3].

TTS predominantly affects postmenopausal women, with approximately 90% of cases occurring in females, particularly those older than 50 years of age [3]. Epidemiological studies estimate that TTS accounts for about 2% to 3% of all patients presenting with symptoms of acute coronary syndrome (ACS), rising to 5% to 6% among female patients [4]. Clinically, TTS mimics myocardial infarction (MI), presenting with acute-onset chest pain, dyspnea, and syncope [3].

Triggers for TTS are broadly categorized into emotional and physical stressors, with physical triggers being slightly more frequent than emotional ones [3]. Pathophysiological mechanisms proposed for TTS include catecholamine-induced myocardial injury, endothelial dysfunction, oxidative stress, and failure of coronary vasomotor regulation [5]. Estrogen deficiency is also hypothesized to play a role in the heightened susceptibility observed in postmenopausal women [5].

Diagnosis of TTS is primarily based on the Mayo Clinic Criteria [6], which includes:

1. Transient hypokinesis, akinesis, or dyskinesis of the left ventricular mid-segments with or without apical involvement; the regional wall motion abnormalities extend beyond a single epicardial vascular distribution; a stressful trigger is often, but not always, present.
2. Absence of obstructive coronary disease or angiographic evidence of acute plaque rupture.
3. New electrocardiographic abnormalities (ST-segment elevation and/or T-wave inversion) or modest elevation in cardiac troponin.
4. Absence of pheochromocytoma and myocarditis.

Despite its reputation as a reversible condition, TTS can lead to severe complications such as acute heart failure, left ventricular outflow tract obstruction (LVOTO), mitral regurgitation, stroke, ventricular arrhythmias, and intraventricular thrombus formation [7].

This review article aims to provide an overview of the long-term survival of patients with TTS, highlighting potential risk factors and comorbidities that may worsen prognosis. It also explores the risk of recurrence, the utility of advanced imaging modalities for prognosis assessment, and the implications of chronic therapeutic strategies in the long-term management of TTS patients.

## 2. Methods

A non-systematic literature search was conducted using PubMed and Google Scholar to identify the most relevant English-language publications from 1990 to 2025, with a focus on articles published between 2015 and 2025. This search aimed to gather information on the long-term prognosis, biomarkers, imaging, risk of recurrence, and long-term management of TTS, including case–control studies, case reports, and review articles, as well as clinical guidelines and consensus documents. The keywords used in the search included “TTS”, “takotsubo syndrome”, “long-term prognosis”, “long-term management”, “risk of recurrence”, “recurrence”, “biomarkers”, “cardiac biomarkers”, “imaging modalities”, “imaging”, and “mortality”. Boolean operators (AND/OR) were used to ensure comprehensive retrieval of articles. References were selected based on their clinical relevance and their contribution to enhancing the understanding of long-term prognosis in TTS patients. It is important to note that no structured inclusion or exclusion criteria, quality assessment tools, or Preferred Reporting Items for Systematic reviews and Meta-Analyses (PRISMA) methodologies were applied in this search.

## 3. Risk Factors and Comorbidities Negatively Affecting Long-Term Prognosis in TTS

Risk stratification in TTS remains challenging due to its variable presentation patterns and the difficulties in developing a risk stratification model or scale that can be applied to all forms of TTS [8]. Patients with TTS are diverse in age, gender, and comorbidities. It has been established that the nature of the triggering factor plays a critical role in determining the long-term prognosis of patients diagnosed with TTS. Uribarri et al. analyzed 939 patients from the Spanish National Registry (RETAKO)—among the patients with TTS caused by physical factors, the rate of mortality was higher than in patients with emotionally triggered TTS, with the worst survival rate in patients with hypoxia as a trigger factor. Additionally, they also proved that male sex, age > 70 years, LVEF < 30%, shock on admission, and diabetes mellitus are predictors of long-term mortality [9]. Ghadri et al. compared the prognosis between TTS and matched ACS patients with comparable mortality rates (data from the International Takotsubo Registry—InterTAK Registry); however, TTS induced by physical activities, medical conditions, or procedures had higher mortality rates than ACS. They also provided evidence that TTS related to neurologic diseases had the worst clinical long-term outcome. According to these results, they proposed a classification to enable the prediction of the outcome of patients with TTS based on different trigger factors: class I—TTS related to emotional stress, class II—TTS related to physical stress (IIa—TTS secondary to physical activities, medical conditions, or procedures; IIb—TTS secondary to neurologic disorders), and class III—TTS without an identifiable triggering factor. It is essential to highlight that the less favorable prognosis of TTS patients with physical triggering factors may be related to their poorer overall health and underlying conditions, and TTS itself might not directly lead to death in these patients [10]. In contrast, other clinical data have shown that patients with TTS may experience a lower incidence of serious in-hospital complications compared to those with ST-segment elevation myocardial infarction, suggesting a potentially more favorable short-term clinical course in selected TTS populations [11].

It is a known fact that the prevalence of reduced LVEF is higher in patients with TTS than in those with ACS [3]. Several studies have proved the connection between severely reduced LVEF and the worse long-term prognosis of patients with TTS. The observational study, conducted on 326 patients from the Takotsubo Italian Network, reported significantly higher in-hospital complications and overall mortality rate in patients with LVEF < 35% on admission, as well as a higher rate of adverse cardiac events during the long-term follow-up, despite the resolution of LV-wall motion abnormalities [12]. Patients with reduced LVEF also presented signs of diastolic dysfunction, which has also been proven to worsen long-term outcomes in patients with TTS [13]. Abumayyaleh et al. revealed that, among the patients with LVEF < 35%, there was a comparable short-term mortality rate in those with TTS and patients with ACS. However, the long-term mortality was significantly higher in TTS and was primarily related to non-cardiovascular causes [14]. TTS is usually a self-limiting disease with spontaneous recovery of wall-motion abnormalities. As factors that can affect the resolution function of LV, Almendro-Delia et al. reported older age, history of neurological disorders, concomitant coronary artery disease, active cancer, physical triggers, elevated inflammatory biomarkers, cardiogenic shock, and lower LV ejection fraction at admission. Patients with TTS and prolonged (exceeding 10 days) recovery had a significantly higher death rate during the 4-year follow-up [15].

Atrial fibrillation (AF) is the most frequent arrhythmia in patients with TTS, and its prevalence is estimated at 5–15% [16]. The most recent studies indicate that the diagnosis of AF is significantly associated with higher long-term mortality in patients with TTS [17,18,19]. It was reported that patients with AF presented higher levels of inflammatory markers, suggesting that the inflammation process plays a vital role in developing this arrhythmia [17,19]. Ventricular arrhythmias occur in 4–9% of patients during a TTS episode and, in 4–6% of cases, lead to cardiac arrest [16]. In an extensive observational study including data from the National Inpatient Sample, the prevalence of ventricular tachycardia (VT) was 4.8%, with identified VT predictors such as concomitant AF, congestive heart failure, and coagulopathy. Diagnosis of VT is connected to higher mortality among these patients. Female sex was associated with a lower risk of VT in patients with TTS and lower mortality [20]. Male sex may be linked to higher mortality rates in TTS, likely due to a greater prevalence of physical triggering factors and severe comorbidities in men [16,21,22]. Additionally, men who experience TTS episodes tend to face more significant socioeconomic disadvantages [23]. Studies have shown that men with TTS are more likely to live alone compared to women and tend to be younger [22]. Although LVEF was similar between the sexes on admission and Days 3 and 5 of hospitalization, it appeared to be lower in men at discharge, despite a similar length of hospital stay [22].

The QT interval prolongation in patients with TTS, which can sometimes occur 72 h after admission, may increase the risk of in-hospital torsade de pointes [24,25]. It is essential to emphasize the role of the serial electrocardiograms (ECGs) that need to be performed during the hospitalization of patients with TTS. Prolonged QTc intervals at admission in patients with TTS may indicate a higher risk of cardiovascular rehospitalization during follow-up [26]. According to the results from the RETAKO registry, a prolonged QT interval was significantly connected with all-cause death and nonfatal cardiovascular events in patients with TTS [27]. However, Pinho et al. recently showed that the QT interval prolongation had no significant impact on in-hospital and long-term outcomes in their study group of patients with TTS [28].

Thangjui et al. conducted a study including 154,919 patients with atrial fibrillation, among whom, 42 developed TTS following electric cardioversion. In this group of patients, diabetes mellitus appeared to be protective against the development of TTS [29]. However, data from the Tokyo Cardiovascular Care Unit Network Registry do not corroborate this observation [30]. Hyperglycemia may be linked to a pro-inflammatory response, and patients with TTS who experience hyperglycemia upon admission may face a poorer prognosis during follow-up [31].

Of 1071 patients with TTS from the German Italian Spanish Takotsubo (GEIST) registry, 30% presented with dyspnea on admission. Arcari et al. identified diabetes, lower LVEF, concomitant pulmonary disease, and atrial fibrillation as factors associated with dyspnea on admission. Patients presenting with this symptom had a greater risk of pulmonary edema, cardiogenic shock, and death, including higher long-term mortality [21]. According to the InterTAK registry results, 7% of patients with TTS presented with acute pulmonary triggers, including exacerbations of asthma/chronic obstructive pulmonary disease (COPD), acute respiratory infections, or acute respiratory distress syndrome (ARDS). The presence of such a triggering factor was associated with worse long-term outcomes. However, concomitant asthma or COPD in patients with non-pulmonary-induced TTS had no impact on the long-term mortality of those patients [32]. Recently, Arcari et al. demonstrated an increased risk of in-hospital complications, recurrent TTS, and long-term mortality in patients with TTS and concomitant COPD. Approximately one-third of these patients presented with acute pulmonary triggers [33].

One of the most common comorbidities among patients with TTS is cancer, and its prevalence in this group is reported to be higher than in the general population. Research has shown that this additional diagnosis significantly increases the risk of in-hospital complications, mortality during hospitalization, and long-term all-cause mortality in these patients [34,35,36].

Lastly, it is crucial to address the psychiatric conditions observed in up to 50% of TTS patients, as these conditions contribute to a greater risk of the development and recurrence of this disease [37]. TTS is most frequently described among postmenopausal women, and both older age and female sex are independent risk factors for certain psychiatric illnesses. The Inter-TAK Registry has reported a higher prevalence of depression and anxiety disorders among TTS patients compared to those with ACS [3]. A similar finding was noted in a study utilizing the National Inpatient Sample, which included over 24,000 patients, providing a higher rate of anxiety and mood disorders in TTS patients compared to controls [38]. In a systematic review conducted by Carroll et al., depression (39%) and anxiety (17%) were identified as the most commonly reported psychiatric illnesses among TTS patients [37]. Additionally, TTS patients who experience recurrence often have a higher frequency of psychiatric medication usage [39]. Moreover, changes in pharmacological therapy can also act as triggering factors for TTS. Tan et al. analyzed data from the Food and Drug Administration Adverse Event Reporting System (FAERS) database and identified 132 cases of TTS related to the use of selective serotonin reuptake inhibitors (SSRIs) and serotonin–norepinephrine reuptake inhibitors (SNRIs). Among these cases, venlafaxine and fluoxetine were the most commonly used antidepressants. However, the exact underlying mechanism remains unclear [40]. Most cases of potentially drug-induced TTS reported in the literature were linked with rapid uptitration or overdose of SNRIs [41,42,43]. A summary of the studiesis presented in Table 1.

**Table 1:** Risk factors and comorbidities negatively affecting long-term prognosis in TTS—a summary of cited studies.

Authors	Year of Publication	Country of Research and/or Used Database	Results
El-Battrawy et al. [18]	2017	Germany	- 114 TTS patients - in-hospital mortality, 30-day mortality, and long-term mortality were significantly higher in patients with AF
Santoro et al. [26]	2017	Germany, Italy	- 52 TTS patients - prolonged QTc interval at admission was significantly associated with a higher risk of rehospitalization at follow-up
Sun et al. [13]	2017	China, United States	- 205 TTS patients - diastolic dysfunction as a risk factor for worse long-term outcomes
Ghadri et al. [7]	2018	International (Austria, Finland, France, Germany, Italy, Poland, Switzerland, the United Kingdom, and the United States)—InterTAK Registry	- 455 TTS patients vs. 455 ACS patients—overall similar long-term mortality - among 1613 TTS patients: worst outcome in patients with TTS caused by neurologic diseases, most favorable prognosis in TTS related to emotional stress - TTS secondary to physical activities, medical conditions, procedures, and neurologic disease: significantly higher mortality compared with ACS
Thangjui et al. [29]	2023	United States—National Readmission Database	- among 154,919 patients admitted with atrial fibrillation, who underwent electrical cardioversion in 2018, 0.027% were readmitted with takotsubo cardiomyopathy - diabetes mellitus seemed protective against developing TTS
Citro et al. [12]	2019	Italy—Takotsubo Italian Network	- 326 TTS patients - LVEF < 35% on admission: higher rate of in-hospital complications, as well as higher overall mortality and rate of adverse cardiac events during the long-term follow-up
Jesel et al. [17]	2019	France	- 214 TTS patients - AF is significantly associated with poorer short- and long-term outcomes
Uribarri et al. [9]	2019	Spain—Spanish National Registry (RETAKO)	- 939 TTS patients—higher mortality in cases of TTS triggered by physical factors(worse prognosis—hypoxia) - other risk factors for less favorable outcome: age > 70 years, diabetes mellitus, LVEF < 30%, shock on admission
Abumayyaleh et al. [14]	2020	Germany	- 648 patients with TTS or ACS - LVEF < 35%: comparable short-term mortality rate in those with TTS and patients with ACS - significantly higher long-term mortality in TTS, but related to non-cardiovascular causes
Budnik et al. [22]	2020	Poland	- 232 patients: 211 women and 11 men - men: more likely to live alone, more frequent physical trigger, higher prevalence of smoking in men, lower LVEF
Carroll et al. [37]	2020	United States	- a systematic review: 252 TTS patients - depression (39%) and anxiety (17%)were identified as the most commonly reported psychiatric illnesses among those patients
Madias et al. [39]	2020	United States	- 128 TTS patients, 117 (91.4%) female, with 47 (36.7%) patients having a neurological and/or psychiatric comorbidity(s) - a higher frequency of psychiatric medication usage
De Miguel et al. [27]	2021	Spain—Spanish National Registry (RETAKO)	- 246 TTS patients - corrected QT interval was independently associated with the primary endpoint of all-cause death and nonfatal cardiovascular events
El-Battrawy et al. [19]	2021	International—InterTAK Registry	- 112 patients with AF (7.1%) out of the overall 1584 TTS patients - among those with AF: higher mean age, fewer women, lower LVEF, more often observed cardiogenic shock, higher in-hospital and long-term mortality
Paolisso et al. [31]	2021	Italy	- 28 patients with TTS and hyperglycemia vs. 48 with normoglycemia - patients with TTS and hyperglycemia exhibit sympathetic overactivity with a hyperglycemia-mediated proinflammatory pathway, which could cause a worse prognosis during follow-up
Arcari et al. [21]	2022	Germany, Italy, Spain—German Italian Spanish Takotsubo (GEIST) Registry	- 286 men (11%) out of 2492 TTS patients - men: younger age, higher prevalence of comorbid conditions (diabetes mellitus, pulmonary diseases, malignancies) and physical trigger; higher rates of cardiogenic shock, in-hospital mortality, and long-term mortality - risk factors for dyspnea on admission: diabetes, lower LVEF, concomitant pulmonary disease, AF
Osawa et al. [34]	2023	Japan	- a systematic review of 14 studies (189,210 TTS patients) - patients with TTS and malignancy: higher risk of mortality at the most extended follow-up; cancer was significantly associated with an increased risk of in-hospital or 30-day mortality
Almendro-Delia et al. [15]	2024	Spain—Spanish National Registry (RETAKO)	- 1463 TTS patients - predictors of late recovery: older age, history of neurological disorders, concomitant coronary artery disease, active cancer, physical triggers, elevated inflammatory biomarkers, cardiogenic shock, lower LVEF at admission
Elkattawy et al. [20]	2024	United States—National Inpatient Sample (NIS)	- 1923 patients with VT (4.8%) out of 40,114 patients - identified VT predictors: concomitant AF, congestive heart failure, coagulopathy - diagnosis of VT was connected to higher mortality among those patients - the female sex was associated with a lower risk of VT in patients with TTS and lower mortality
Palm et al. [23]	2024	Denmark—Danish national registries	- 79 men (11.9%) out of 662 TTS patients - men: socioeconomically disadvantaged compared to their female counterparts, with higher 3-year mortality after an incident (most pronounced among men with a low family income and an increase in comorbidity score)
Pinho et al. [28]	2024	Portugal	- 113 TTS patients - QT interval prolongation had no significant impact on in-hospital and long-term outcomes
Tan et al. [40]	2024	International—FAERS	- 132 cases of TTS related to SSRIs and SNRIs therapy (female patients—80%)
Tini et al. [35]	2024	Italy	- a systematic revision of 1109 TTS cases - 10% mean prevalence of cancer in TTS, ranging from 4% to 29%
Watanabe et al. [30]	2024	Japan—Tokyo Cardiovascular Care Unit Network Registry	- 1226 TTS patients - the prevalence of diabetes was 17.0% in TTS and 15.8% in the general population, with no significant difference - patients with diabetes tended to have a higher in-hospital mortality rate
Arcari et al. [33]	2025	Italy, United Kingdom	- 69 patients with TTS and COPD out of the overall 440 patients - COPD: higher risk of in-hospital complications, long-term recurrence, and mortality

## 4. Biomarkers and Imaging in TTS

Coronary angiography and left ventriculography are considered the gold standards for the acute evaluation of suspected TTS and for differentiating it from ACSs, including acute myocardial infarction (AMI). These procedures are essential for ruling out obstructive coronary disease and identifying typical wall motion abnormalities. All patients presenting with acute chest pain and changes on their ECG should undergo a rapid assessment for ACS. Typically, patients with TTS demonstrate normal coronary arteries or mild atherosclerosis, distinguishing it from AMI, where significant blockages are often present [44]. Ventriculography, performed in conjunction with angiography, usually reveals the classic apical ballooning pattern, although midventricular, basal, and focal variants are also recognized [7].

Additional imaging techniques, including computed tomography (CT), nuclear imaging (single-photon emission computed tomography (SPECT) and positron emission tomography (PET)), and sympathetic innervation imaging, have also been applied in TTS research, revealing persistent sympathetic denervation in affected segments [7]. CT angiography can be a non-invasive alternative for coronary evaluation, while PET imaging has been investigated for assessing myocardial perfusion and metabolic activity during TTS episodes [44]. These modalities provide supplementary insights into the pathophysiological changes that occur during the acute and recovery phases of TTS.

The usefulness of cardiac biomarkers is evident in situations where the diagnosis is unclear. TTS presents a distinct biochemical and imaging profile that facilitates its differentiation from ACS, especially ST-segment elevation myocardial infarction (STEMI), in diagnostically ambiguous presentations, or as an adjunctive evaluation strategy [44]. Troponin levels, usually equally elevated on admission compared to ACS, have substantially lower peak values than classical ACS [3,7]. Templin et al. demonstrated a mean 1.8-fold increase in troponin levels in TTS compared to an approximately six-fold rise over admission values in STEMI [3]. Meta-analysis confirms this disparity, showing that peak troponin levels in ACS exceed those in TTS by roughly 75 times the upper limit of normal [44]. Moreover, an initial troponin concentration exceeding 10 times the upper limit of the normal level was associated with a higher incidence of the combined endpoint, including in-hospital death [3]. A combination of peak troponin I levels and echocardiographically measured LVEF has recently been proposed as a diagnostic index to differentiate TTS from STEMI, reflecting the disproportion between troponin elevation and the extent of ventricular dysfunction in TTS. A threshold value of ≥250 demonstrates a sensitivity of 95% and specificity of 87% for distinguishing TTS from STEMI [45].

Conversely, NT-proBNP levels are markedly elevated during the acute phase of TTS [7,16]. These biomarkers often rise to levels 5.9 times the upper reference limit, significantly exceeding those found in ACS patients [3,16,46]. BNP and NT-proBNP concentrations correlate with the extent of ventricular wall motion abnormalities and catecholamine excess [16,46]. Importantly, NT-proBNP levels peak within 24–48 h of symptom onset and then gradually return to normal over the subsequent weeks to months [7]. The existing literature supports the superior diagnostic utility of natriuretic peptides compared to troponins in TTS, and it is recommended that they be measured in all suspected cases if the assay is available [46].

Several biomarkers are promising in differentiating TTS from MI. The serum copeptin/NT-proBNP shows high diagnostic accuracy, as copeptin levels are significantly lower in TTS compared to STEMI and could be an additional tool for non-invasive differentiation [47]. Likewise, NT-proBNP/myoglobin and NT-proBNP/troponin T ratios effectively distinguish TTS from both STEMI and NSTEMI [48]. Recent findings also show that NT-proBNP/TnI, NT-proBNP/CK-MB mass, and NT-proBNP/EF ratios are significantly higher in TTS than in STEMI, with the NT-proBNP/TnI ratio demonstrating the most significant diagnostic accuracy, thereby offering valuable metrics for early differentiation [49]. Novel biomarkers, including growth differentiation factor-15 (GDF-15), are elevated in TTS compared to STEMI and have prognostic value, particularly in patients with biventricular ballooning [50]. Additionally, inflammatory markers such as IL-6 and IL-7 exhibit distinct profiles in TTS versus MI, with lower IL-6 levels and higher IL-7 levels in TTS [51]. Furthermore, circulating microRNAs also show promise; elevated circulating levels of miR-1, miR-16, miR-26a, and miR-133a create a distinct signature that can differentiate TTS from STEMI with up to 97% sensitivity [52,53].

Regarding imaging, echocardiography is the most widely used imaging modality due to its accessibility and utility in detecting regional wall motion abnormalities, LVOTO, mitral regurgitation, and apical thrombus [7,45]. Advanced echocardiographic methods, including speckle-tracking echocardiography and strain imaging, further enhance diagnostic accuracy and can monitor the recovery of ventricular function [45,54]. LV function typically recovers within 4–8 weeks, with serial echocardiography recommended to track functional improvement [45,54]. Echocardiography can also detect acute complications such as LV rupture or intracardiac thrombi [7,55].

Cardiac magnetic resonance imaging (MRI) provides high-resolution tissue characterization and has become a cornerstone in the subacute assessment of TTS. Moreover, it is used to differentiate it from myocardial infarction and myocarditis. In addition to the identification of typical symmetric regional wall motion abnormalities, cardiac MRI allows precise evaluation of RV and LV function, assessment of additional complications (i.e., pericardial and/or pleural effusion, LV and RV thrombi), and characterization of myocardial tissue (i.e., oedema, inflammation, necrosis/fibrosis) [7]. Recently, specific cardiac MRI criteria for TTS diagnosis at the time of acute presentation were established, including the combination of typical symmetric regional wall motion abnormalities, edema, and the absence of evidence of irreversible tissue injury (late gadolinium enhancement (LGE)) [7]. Notably, the absence of LGE in dysfunctional LV regions allows distinction between TTS and other conditions, including ACS (subendocardial or transmural LGE corresponding to a vascular territory) and many cases of acute myocarditis [7]. It reflects a lack of irreversible myocardial injury [7,56,57]. However, some studies show that transient LGE may occur and typically resolves within 12 months [56]. Myocardial edema, visible on T2-weighted imaging and mapping techniques, is diffuse, not restricted to a vascular territory, and gradually normalises over weeks, distinguishing it from AMI and myocarditis [57]. In AMI, the oedema persists for weeks [58,59]. Additionally, in TTS, the edema is diffuse and transmural, whereas myocarditis is more commonly seen in the subepicardial or mid-myocardial region [57]. Cardiac MRI is also valuable for detecting subtle fibrosis, RV involvement, and assessing myocardial perfusion, particularly in subacute and follow-up phases [7,57]. Quantitative perfusion imaging and T1/T2 mapping can objectively evaluate myocardial injury and microvascular dysfunction [57]. It also serves as the gold standard for the follow-up of these patients after 3–6 months to establish reversibility, which is the hallmark of TTS [57].

**Figure 1:**
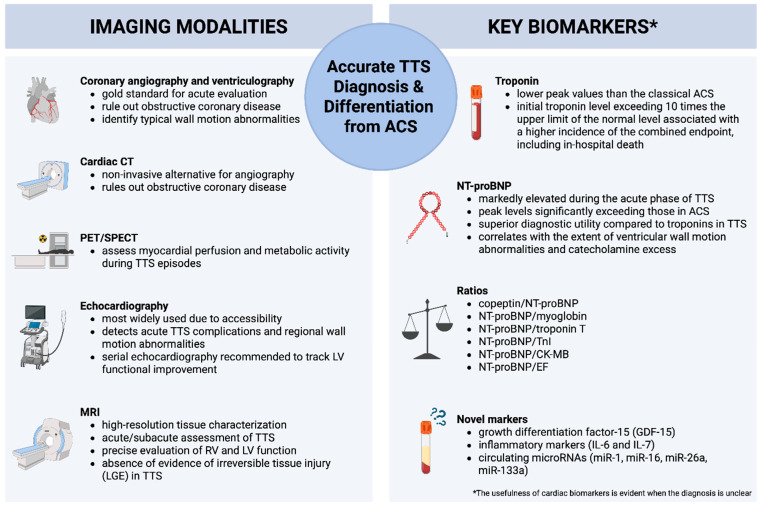
Imaging modalities and key biomarkers in TTS. TTS—Takotsubo syndrome, LGE—late gadolinium enhancement, CT—computed tomography, PET—positron emission tomography, SPECT—single photon emission computed tomography, LV—left ventricle, RV—right ventricle, ACS—acute coronary syndrome. Created in https://BioRender.com.

In summary, integrating both conventional and advanced biomarkers, along with multimodal imaging strategies, enhances the diagnostic accuracy and clinical management of TTS (Figure 1). These tools not only help differentiate TTS from AMI, but also contribute to evaluating myocardial injury, assessing ventricular function, and predicting long-term outcomes in affected patients.

## 5. Recurrence of TTS

Recurrence of TTS is relatively uncommon; however, it represents a significant clinical concern. The recurrence rates in TTS vary across different studies, and knowledge of long-term TTS complications is limited due to the relatively small number and short follow-up periods in most cohorts [60]. Meta-analyses of 31 studies report a cumulative incidence of recurrence ranging from 1.2% during the first 6 months to nearly 5% at 6 years, while data from the multicenter GEIST registry estimate the cumulative TTS recurrence risk at 4% over a median follow-up period of 2.3 years [60,61]. However, some studies report variations from 3.2% to 11.4%, with a median time to recurrence of approximately 2–3 years, and extreme cases ranging from as early as 3 weeks to as late as 13.8 years after the initial event [6,7,8,9,11,12]. Annualized recurrence rates range from 1% to 2% [3,60,61,62,63].

The majority of patients experiencing recurrence typically have only one additional episode (approximately 90%), though cases with multiple recurrences are also observed. Lau et al. describe three patients with two episodes of recurrence (7.7%) and one patient with three episodes of recurrence (2.6%) among 39 recurrent TTS cases [62]. Shaw et al. describe this phenomenon as “super recurrence,” defined as two or more episodes, and characterize it as affecting approximately 2% of patients with TTS [64]. Patients with super recurrence tend to be younger, with significantly higher peak troponin, a lower ejection fraction, and a higher prevalence of emotional triggers, depression/anxiety, and cancer diagnoses [64]. The combination of a lower ejection fraction and greater peak troponin suggests that patients with super recurrence experience more severe cardiac injury during TTS events, although this scenario has not been thoroughly examined [64]. A meta-analysis of 121 studies involving 128 patients with recurrent TTS demonstrated that 24.2% of these patients experienced multiple recurrence events, ranging from two to a maximum of five episodes [39].

Sex, age, and comorbidities appear to influence the risk of recurrence. In a study by Lau et al., male sex was associated with a 2.5-fold higher risk of recurrence or death compared to females [62]. Notably, Patel et al. found a 5-fold higher recurrence rate in female patients under 50 years of age compared to older women [65]. Other independent risk factors for recurrence also include increasing age, diabetes, chronic kidney disease, and pulmonary disease [62]. Additionally, the GEIST Registry identified significantly higher rates of arterial hypertension and chronic obstructive pulmonary disease/asthma in the recurrence group, corroborating findings from El-Battrawy et al. [61,66].

The influence of pharmacological therapy remains a matter of controversy. Some studies indicate that beta-blocker therapy reduces the risk of recurrence and mortality compared to those not treated with beta-blockers [60], while others found no significant association [60,64,67]. Moreover, Templin et al. found that one-third of patients experienced a TTS recurrence during beta-blockade [3], suggesting that other receptors, such as alpha-receptors, that are more prevalent in the coronary microcirculation, might be involved [7]. Angiotensin-converting enzyme inhibitors (ACEi) or angiotensin receptor blockers (ARBs) have shown a protective effect against recurrence [60], although some studies report no such association [39]. Combined therapy with beta-blockers and ACE inhibitors or ARBs appears promising in reducing recurrence risk [36,68,69].

Regarding clinical presentation, the apical type of TTS is the most common during recurrence, although midventricular forms are also frequent [39,61]. The kind of TTS may vary between episodes; while the apical form predominates, midventricular or right ventricular ballooning occurs in 20–30% of recurrent cases [39,61,68,70]. A case report by Ghadri et al. describes a patient with three TTS episodes, each with distinct wall motion patterns (mid-ventricular, apical, and focal type), highlighting the variability in presentation [17].

LV function also plays a critical role. Meta-analyses indicate that patients with recurrence have a lower average LVEF during the first episode (33%) compared to the non-recurrence group (40%), suggesting that initial severity may predict recurrence risk [39,62]. Topf et al. observed that cardiovascular biomarkers, like pro B natriuretic peptide (proBNP) and high-sensitivity troponin levels, varied between recurrent and non-recurrent groups, although only the initial LVEF showed a significant difference [39]. However, data from the InterTAK Registry did not reveal substantial differences between the two groups regarding LVEF [61]. A lower LVEF during the initial TTS episode may indicate greater myocardial vulnerability or severe initial cardiac injury and, as a result, be associated with a higher recurrence risk [39,62].

Symptoms at recurrence typically mirror those of the initial event, with chest pain and dyspnea being predominant. However, trigger types often differ between episodes, with approximately 40% to 67% of patients experiencing new emotional or physical triggers at recurrence [61,64,68]. Data from the RETAKO Registry revealed that patients with recurrent TTS, compared to those with no recurrence of TTS, were more likely to have no identifiable stressful trigger at the index event [71]. Primary TTS, defined as TTS without a physical trigger, was also more common in the recurrence group [71]. This supports the hypothesis that some patients may have heightened intrinsic susceptibility to TTS in the absence of a specific stressor.

Importantly, recurrence has significant prognostic implications. One study found that TTS patients with recurrence had a 5.9-fold higher 30-day cardiovascular mortality rate compared to non-recurrent cases [67]. Fatal arrhythmias, such as ventricular tachycardia, occurred significantly more often in the recurrence group [67]. However, long-term survival may not differ significantly between recurrence and non-recurrence groups, particularly in patients who recover LVEF [66,72]. There was no significant difference regarding different in-hospital events such as thromboembolic events, stroke, cardiogenic shock, and the use of cardiac electronic device implantation. However, pulmonary edema was more often documented in the recurrence group than in the non-recurrence group at the index event, though in-hospital mortality and hospital stay duration did not differ between the two groups [61].

Finally, while a potential genetic predisposition to recurrence has been hypothesized, current data remain inconclusive. Familial forms and repeated recurrences suggest a possible genetic predisposition that interacts with environmental triggers and adrenergic dysregulation [36]. Given the complexity and variability in recurrence patterns and presentations, ongoing research into recurrence risk factors, preventive therapies, and potential genetic influences remains essential to improving patient outcomes and clinical management.

## 6. Long-Term Survival of Patients with TTS in Numbers

This summary presents the most recent data on mortality rates among patients with TTS (Table 2). According to the results from the InterTAK Registry, published in 2015, the rate of death among 1750 patients with TTS was 5.6% per patient-year [3]. The results presented in 2024 showed that out of 2938 patients, 7.6% (222) died during a 1-year follow-up [73]. The observational study performed by Stiermaier et al. revealed that 286 TTS patients, compared to an equal number of patients with STEMI, matched for age and gender, presented with significantly higher mortality during the follow-up period (mean of 3.8 ± 2.5 years), although this was primarily driven by non-cardiovascular causes [74]. The SWEDEHEART (Swedish Web-system for Enhancement and Development of Evidence-based care in Heart disease Evaluated According to Recommended Therapies) registry results indicated that long-term mortality rates for patients with TTS were comparable to those of ACS [75]. Regarding another observational study, in which Butt et al. analyzed data from Danish nationwide registries from 2011 to 2018, patients with a history of TTS also had a higher associated death rate compared to patients with STEMI (absolute 3-/6-year risk of death 19.7%/29.4% and 11.3%/21.8%, respectively). Moreover, the investigators compared the patients with TTS to the selected general population; in this comparison, survivors of TTS also had a higher rate of death. One of the secondary outcomes was hospitalization due to heart failure (HF)—the risk of HF hospitalization among patients with TTS was higher than the corresponding risk in the general population, but significantly lower than in patients with STEMI [76]. Pelliccia et al. conducted a meta-analysis of 54 studies with follow-up data from more than 6 months. Of the total 4679 patients diagnosed with TTS, the death rate during admission was 2.4% (112 patients). The annual mortality rate of the other 4567 patients was 3.5% (with a mean follow-up of 28 months), and the majority of these patients died because of non-cardiac reasons. Factors that negatively impacted long-term prognosis in each study included older age, physical stressors, and atypical patterns of wall motion abnormalities associated with TTS [77]. In a smaller observational study conducted on a group of TTS patients, the majority presented with physical triggering factors, providing a mortality rate of 39.1% [78].

**Table 2:** Long-term survival of patients with TTS in numbers—a summary of cited studies.

Authors	Year of Publication	Country of Research and/or Used Database	Results
Redford et al. [75]	2015	Sweden—SWEDEHEART Registry, Swedish Coronary Angiography and Angioplasty Registry (SCAAR), and the Register of Information and Knowledge about Swedish Heart Intensive Care Admissions (RIKS-HIA)	Short- and long-term mortality in patients with Takotsubo (*n* = 302) and patients with ST-elevation myocardial infarction (STEMI, *n* = 6595) and non-ST-elevation myocardial infarction (NSTEMI, *n* = 8207) were similar
Templin et al. [3]	2015	InterTAK Registry	- mortality among 1750 patients with TTS was 5.6% per patient-year
Stiermaier et al. [74]	2016	Germany	- 286 TTS patients matched for age and gender with 286 STEMI patients - significantly higher long-term mortality compared with the matched STEMI cohort (24.7% vs. 15.1%) during the follow-up period (mean of 3.8 ± 2.5 years; of note—primarily driven by non-cardiovascular causes)
Pelliccia et al. [77]	2019	meta-analysis of 54 studies with follow-up data exceeding 6 months	- 4679 TTS patients: the death rate during admission was 2.4% (112 patients); the annual mortality rate of the other 4567 patients was 3.5% (with a mean follow-up of 28 months), and the majority of those patients died because of non-cardiac reasons
Gaede et al. [78]	2020	Germany	- 126 TTS patients - the majority presented with physical triggering factors: a mortality rate of 39.1% within 4 years
Butt et al. [76]	2022	Denmark—Danish nationwide registries	- 881 TTS patients - mean follow-up of 2.9 years - survivors of TTS had higher associated rates of death compared with the background population - survivors of TTS had higher associated rates of hospitalization due to HF compared with the background population, but lower rates compared with survivors of STEMI
Stähli et al. [73]	2024	InterTAK Registry	- out of 2938 patients, 7.6% (222) died during a 1-year follow-up.

## 7. Acute Treatment in TTS

Management of the acute phase of the disease depends on the severity of the heart failure and the identified risk of complications. Ideally, all patients should be admitted to the intensive care unit (ICU) and monitored with an ECG for at least 24 h. In the past, beta-blockers were commonly used in the acute phase of TTS; however, their beneficial effects have not been proven to date [79]. Specific complications may require the administration of antiarrhythmics, non-catecholamine inotropics (such as levosimendan), and anticoagulants [16]. Based on findings from the GEIST Registry, prophylactic oral anticoagulants (OACs) should be considered in cases with a risk of late intraventricular thrombus formation, particularly those with an apical ballooning pattern and elevated troponin I levels exceeding 10 ng/mL upon admission [80]. Extensive segmental akinesia may also be a reason for implementing anticoagulant prophylaxis [16]. For patients with TTS complicated by cardiogenic shock, it is crucial to consider mechanical circulatory support, such as extracorporeal membrane oxygenation (ECMO) or microaxial pumps (e.g., Impella) [79].

## 8. Long-Term Management and Chronic Treatment of Patients with TTS

As part of the long-term care of patients with TTS, a follow-up visit is recommended after 3 to 6 months. This visit should include an ECG and cardiac imaging, such as transthoracic echocardiography, as well as possibly an MRI [16]. Since one of the most common triggers for TTS is emotional stress, there may be a role for psychological counseling and psychiatric assessment of these patients [81].

The available data on the long-term treatment of patients with TTS are primarily based on several observational studies, as there are no completed randomized trials on the chronic pharmacological treatment of these patients [79]. Omerovic et al. have initiated the first ongoing randomized trial, BROKEN-SWEDEHEART (Sweden), which aims to evaluate the effects of treatment with adenosine and dipyridamole on cardiac recovery in patients with TTS. The study will include 1000 patients and investigate the role of apixaban in reducing the risk of thromboembolic events [82]. According to the InterTAK registry results, angiotensin-converting-enzyme inhibitors (ACEi) or angiotensin-receptor blockers (ARBs) improve the 1-year survival of patients with TTS. However, the use of beta-blockers was not associated with such an outcome, despite their effectiveness against stressful triggers and catecholamine surges [1,83]. Most recent analyses based on the RETAKO registry also showed no association with using beta-blockers and lower mortality or risk of TTS recurrence. Moreover, they did not prove the former evidence on the potential beneficial impact of beta-blockers in patients who experienced cardiogenic shock during TTS [83,84]. D’Ascenzo et al. (InterTAK Registry) proved that chronic aspirin therapy in TTS is not associated with a reduced risk of major adverse cardiac and cerebrovascular events at 30-day and 5-year follow-ups [85]. A systematic review and meta-analysis conducted by Rizzetto et al., including several more studies, supported this outcome and found statistically significant proof of the negative impact of antiplatelet therapy (APT) on long-term prognosis and mortality in TTS patients [86]. However, recent results from the RETAKO registry suggest that the use of APT may be related with better long-term outcome in the first 25 months of follow-up for patients with TTS [87]. The indications for APT in these patients require further investigation. Analyses based on the GEIST registry revealed no correlation between statin treatment and the improved long-term prognosis of patients with TTS [88]. Additionally, recent findings from the SWEDEHEART registry suggest a possible link between the use of heparin and statins with lower 30-day mortality, as well as between statins and ACE inhibitors with reduced long-term mortality. Conversely, there was no statistically significant correlation with mortality and treatment with ARBs, oral anticoagulants, P2Y12 antagonists, aspirin, or beta-blockers [89]. Furthermore, patients with TTS complicated by intraventricular thrombi require chronic anticoagulation therapy to prevent thromboembolic events, with data indicating that this therapy should last for at least three months [80].

## 9. Conclusions

Following initial observational studies, TTS was predominantly considered a benign, self-limiting condition with a favorable long-term prognosis and no significant impact on the survival of patients with TTS. However, the most recent findings have challenged this theory, providing evidence that TTS is a heterogeneous condition with various presentation patterns, and patients’ comorbidities often complicate its clinical course and long-term prognosis. Classically, TTS was perceived as an emotionally triggered disorder; however, it is essential to note that more than one-third of patients present with TTS induced by a physical trigger. Among the most critical determinants adversely affecting the long-term prognosis of TTS patients are pulmonary and neurological diseases. The other factors with an observed negative influence on long-term outcome are: male sex, older age, an initially reduced left ventricular ejection fraction, and dyspnea or shock on admission. Significant comorbidities linked to a poorer long-term prognosis include concomitant coronary artery disease, atrial fibrillation, chronic obstructive pulmonary disease, and active cancer. The influence of diabetes mellitus on long-term prognosis in TTS may seem controversial, but the majority of studies revealed its potential to worsen long-term outcomes. The potential presence of comorbidities and the fact that mortality in TTS, in most studies, was related to non-cardiovascular causes, may justify a multidisciplinary approach for TTS patients. However, since one of the most common triggers for TTS is still emotional stress, there may be a role for psychological counseling and psychiatric assessment of these patients. The combined use of cardiac biomarkers, such as relatively low peak troponin levels, markedly elevated NT-proBNP, and diagnostic ratios including copeptin/NT-proBNP, along with advanced imaging modalities, provides a robust framework for differentiating TTS from acute coronary syndromes. Furthermore, the integration of echocardiography for the early detection of wall motion abnormalities and complications, alongside cardiac MRI for tissue characterization and confirmation of myocardial reversibility, enhances diagnostic accuracy and longitudinal monitoring in patients with TTS. While data on chronic treatment remain limited, further research is needed to identify the most effective pharmacological interventions for patients with TTS, preferably including randomized controlled trials. Those interventions should aim to improve long-term outcomes and decrease the risk of mortality and TTS recurrence.

## Data Availability

No new data were created.
